# Editorial: Advances in CNS Repair, Regeneration, and Neuroplasticity: From Basic Mechanisms to Therapeutic Strategies

**DOI:** 10.3389/fncel.2022.898546

**Published:** 2022-05-27

**Authors:** Shuxin Li

**Affiliations:** Department of Neural Sciences, Shriners Hospitals Pediatric Research Center, Lewis Katz School of Medicine at Temple University, Philadelphia, PA, United States

**Keywords:** CNS injury, brain injury, spinal cord injury, neural repair, axon regeneration

Loss of neural cells and neuronal networks in the CNS frequently results in permanent functional deficits with minimal recovery. Current treatments for patients suffering from neurological functional deficits are largely limited. Recent studies have provided insight into secondary injury mechanisms and signaling pathways for controlling CNS repair and regeneration. Researchers have identified new molecular and cellular targets and effective therapeutic strategies for promoting cell survival, CNS regeneration, neural circuit reconnections, remyelination, and neuroplasticity in adult CNS. This Research Topic focuses on recent preclinical research advances in CNS repair, regeneration, and neuroplasticity. We published 29 articles on this Research Topic, including 17 reviews and 12 original research articles. These studies targeted multiple molecular and cellular components using several model systems, including traumatic brain injury (TBI), spinal cord injury (SCI), optic nerve crush (ONC), neurodegeneration, and *in vitro* cell cultures. Many articles focused on enhancing viability of neural cells and regeneration, reconnection, and plasticity of injured CNS neurons by targeting diverse CNS pathophysiology, including inflammatory cascades and regeneration failure of axotomized CNS axons.

## Neural Repair After Brain Injury

TBI is one of the leading causes of morbidity, disability, and mortality. Recent studies have advanced our further understanding of complex pathogenesis and the development of effective therapeutic strategies. Ng and Lee overviewed the pathophysiology, underlying molecular mechanisms, and new therapeutic targets and agents for TBI. Following the irreversible primary damage to the brain parenchyma, secondary brain injuries start acutely and progress slowly over months to years, including ischemia, hemorrhage, mitochondrial dysfunction, excitotoxicity, oxidative stress, neurodegeneration, and apoptotic/necrotic death of neural cells. Some druggable targets have been identified to intervene with these processes. Certain promising therapeutic approaches had been moved to clinical trials for TBI although most failed to show effectiveness in phase III clinical trials. Shaughness et al. reviewed the therapeutic potential of insulin for neurotrauma and various neurodegenerative disorders. Insulin, a clinical hormone drug, interacts with insulin receptors on neurons and glia and regulates their cellular metabolic and non-metabolic functions. Insulin also suppresses inflammatory responses and promotes neuronal survival and functional recovery after CNS lesions. Given its wide systemic or intranasal use and reported benefits, insulin is promising for translational treatments of various neurological diseases.

O'Reilly et al. reviewed neuroimmune-mediated plasticity after CNS injury. Spontaneous plasticity frequently occurs after neurotrauma, affects multiple neuronal circuits, and may produce beneficial and deleterious functional outcomes largely depending on the context of plasticity and circuits affected. CNS injury activates the neuroimmune system and numerous immune and inflammatory factors, which may alter neuroplasticity at cellular levels. Recent advances in further dissecting the roles of neuroimmune factors may offer important therapeutic options for fostering adaptive plasticity and reducing maladaptive plasticity. Cerqueira et al. updated nanoparticle-based methods for drug delivery to CNS. Efficient drug delivery to CNS is critical for treating neurological diseases. Emerging nanomaterial technologies provide a promising tool to achieve this goal. Biomimetic, cell-targeted, and stimuli-responsive multifunctional nanoparticles have been evaluated to deliver bioactive compounds to treat CNS disorders. The advanced nanoparticle-based delivery may also achieve a spatiotemporally controlled strategy for optimal drug administration to targeted CNS regions.

## Axon Regeneration and Neuroplasticity After CNS Injury

CNS axon injury leads to devastating and persistent functional loss because axotomized CNS axons generally fail to regenerate. Restoring lost functions requires reconnections of injured axons with distal targets and/or the formation of synaptic relays by interneurons. The larger actin-supported extension region of a developing or regenerating axon is referred to as a growth cone. The role of axon growth cone during development is well characterized, but its function in regrowth in response to CNS injury remains an active topic of research. Rodemer, Gallo et al. reviewed the cytoskeletal substructures of axon growth, elongation of axon tip, and potential development of regenerative therapies by targeting the growth cone. This review largely focused on the cytoskeletal dynamics at axon tip underlying regenerative extension.

Traumatic CNS injury, including SCI, results in glial scar formation around the lesion. The extracellular matrix molecules, mostly tenascins and chondroitin sulfate proteoglycans (CSPGs) accumulate in the scar tissues and form an inhibitory barrier to axonal regeneration. Overcoming inhibition by CSPGs and scar tissues may facilitate developing effective strategies for regenerative therapies. Hussein et al. discussed the role of CSPGs in response to CNS injury and suppression of sulfated glysosaminoglycan (GAG) chains on neuronal regeneration and plasticity. This review covers the influences of GAG chain sulfation on their biological activity and the interactions between GAGs and CSPG receptors. CSPGs suppress axon regrowth largely by binding with high affinity to several transmembrane receptors, including two members in the LAR subfamily, LAR and receptor protein tyrosine phosphatase σ (RPTPσ). Multiple intracellular signaling pathways downstream of these two RPTPs mediate the growth-inhibitory actions of CSPGs. Sami et al. reviewed recent advances in the downstream signaling pathways of scar-mediated inhibition and their potential as the molecular targets for CNS repair. A better understanding of these signaling pathways may facilitate the development of new and effective therapies for SCI and other CNS disorders. RPTPσ is one of the CSPG receptors and its deletion or pharmacological blockade has been reported to promote axon regeneration in rodent models. Unexpectedly, using the unique lamprey SCI model, which robustly recovers locomotion after complete spinal cord transection, Rodemer, Zhang et al. reported that RPTPσ knockdown with siRNA impaired regeneration of descending reticulospinal axons 10 weeks after transection primarily due to reduced long-term neuron survival. It will be interesting to further dissect the significance and mechanisms underlying neuronal death after RPTPσ knockdown in SCI lamprey.

After SCI, especially incomplete lesions, spinal interneurons have been reported to be important for mediating neuroplasticity and functional recovery in mammals. Some preclinical strategies have been used to promote neuronal circuits and rewiring by targeting interneurons in the lesioned spinal cord. Zavvarian et al. reviewed the diverse functions of spinal interneurons in regulating pathophysiology after SCI and the potential to treat SCI by targeting interneuron-mediated neuroplasticity. Patients with cervical SCI usually have functional deficits in both upper and lower limbs. Regaining hand function may significantly improve the quality of life in these patients. Gallegos et al. reported that modified Montoya staircase rehabilitation training in C5 SCI rats improved injury environment, axon sprouting and synaptic plasticity, and reaching and grasping functions of the forelimb. Six-week of rehabilitation training started 8 weeks after SCI significantly increased the number of retrieved food pellets, the accuracy of pellet retrievals, and the number of serotonergic fibers and presynaptic terminals around motor neurons in the ventral horns of cervical spinal cord caudal to the lesion, while reduced GFAP immunoreactivity, a marker for astrogliosis. This study further supports the therapeutic potential of task-specific rehabilitation for recovering lost functions after chronic SCI.

Rho family members are critical for regulating cell growth and axon elongation and Clostridium botulinum C3 exoenzyme (C3) is frequently used as a Rho inhibitor. Adolf et al. compared the effects of C3 treatment on neurite growth and branching in sorted mouse GABAergic and glutamatergic neuronal cultures derived from transgenic mice and detected higher sensitivity of GABA neurons to C3 treatment. Analysis of C3 binding partners indicated a higher expression level of vimentin in GABAergic neurons, in contrast to similar levels of beta1 integrin in both neuronal types. Accordingly, C3 showed stronger binding to GABA neurons than to glutamatergic neurons. The different vimentin levels in GABA and glutamatergic neurons appeared to mediate the distinct responses of two types of neurons to C3 treatment. Transplantation of Schwann cells is promising for treating SCI, but grafted Schwann cells usually showed limited migration within an astrocytic environment in the CNS. Li et al. reported enhanced Schwann-astrocytic integration *in vitro* by overexpressing miR-124 in Schwann cells. Overexpressing miR-124 in Schwann cells upregulated various neurotrophic factors (e.g., NT-3 and BDNF) and genes that regulate cell migration, while downregulated GFAP and other cell migration genes. Schwann-astrocytic co-culture assay suggested that miR-124 overexpression in Schwann cells altered the phenotypes of co-cultured astrocytes.

## Secondary Injury and Neuroprotection After SCI

SCI usually causes complicated pathophysiology around the lesion, including neural cell loss, neurodegeneration, glial scar formation, and axon disconnections. Currently, the effective treatments to restore functional loss after SCI are still unavailable. The SCI lesions in most patients are anatomically incomplete. The spared neural pathways provide therapeutic opportunities to enhance anatomical and functional neuroplasticity using rehabilitative, electrophysiological, and pharmacological strategies. High and mid-cervical SCI frequently damages the phrenic motor circuits and neural networks that control respiration and results in diaphragm paresis/paralysis and respiratory deficits. Randelman et al. reviewed the advances of translational pre-clinical strategies for enhancing plasticity and functional outcomes after cervical SCI. Non-invasive respiratory treatments may enhance neuroplasticity and long-term recovery, including clinical use of respiratory training (e.g., resistance training) and pre-clinical and early clinical testing of intermittent chemical stimulation by altering inhaled oxygen (hypoxia) or carbon dioxide. These strategies also have the potential to improve locomotor and other neural networks.

As the major cellular component of the innate immune system in CNS, microglia respond to SCI, regulate neuroinflammation, generate various cytokines and chemokines, clarify cellular debris, and coordinate certain repair processes. Recent studies suggest that the microglial voltage-gated proton channel Hv1 signaling contributes to the pathophysiology of CNS injury, including posttraumatic neuroinflammation and neurodegeneration. He et al. summarized recent progress in Hv1-mediated neural damage, including Hv1-NOX2 (NADPH oxidase 2) interactions to regulate the levels of reactive oxygen species and cytosolic pH. This review also discussed the potential to design a neurotherapy that targets Hv1.

After SCI, neutrophils are the first immune cells that infiltrate the injured spinal cord. Neutrophils appear important for propagating inflammatory responses and recruiting other immune cells, such as macrophages, to the injured area. Zivkovic et al. reviewed the potential roles of infiltrated neutrophils after SCI, including their contribution to the pathophysiology of the injured spinal cord. NG2 (also known as CSPG4) is highly expressed in oligodendrocyte precursor cells, pericytes, and activated macrophages and microglia after SCI, but the significance of NG2+ cells is largely unknown. Liu et al. reported that the infiltrated bone marrow-derived macrophages formed part of NG2 components in the glial scar and contribute to the pathophysiology of injured spinal cord. NG2 was transiently expressed in marrow-derived macrophages in the glial scar region. Myelin debris uptake upregulates NG2 levels in macrophages *in vitro*, suggesting that macrophages that take up myelin debris around CNS lesions may upregulate NG2 in immune cells and alters their phagocytic and proliferative capacity.

Post-SCI treatment with Azithromycin, an FDA-approved macrolide antibiotic, has been reported to reduce pro-inflammatory macrophage activation. A combined pre- and post-injury treatment paradigm improved functional recovery in SCI rodents. Kopper et al. further reported that post-injury Azithromycin treatment improved morphological and functional outcomes in SCI rats. Early Azithromycin treatments beginning 30-min and 3-h post-SCI improved locomotor stepping recovery. The 30-min delayed treatments also reduced pathology of the lesioned spinal cord, in contrast to ineffectiveness of treatment initiated 24-h post-SCI.

Congenital myelomeningocele (MMC), the most common and severe type of spina bifida, frequently causes progressive injury to the exposed spinal cord and lifelong disabilities. MMC results from incomplete neural tube closure and is characterized by a spinal cord and meninge protrusion through a pathological opening in the overlying vertebrae at the lumbosacral region. Janik et al. reviewed the pathophysiology of MMC, *in utero* injury to the exposed spinal cord, and therapeutic options for MMC. Neonates with detected MMC usually receive an early postnatal surgical repair of MMC pathology. Because neural tube closure failure in early gestation initiates progressive prenatal injury to the exposed spinal cord and deteriorates neurological function in fetuses, it is important to develop effective prenatal therapies that prevent continuing spinal cord damage and improve neurological function. *In utero* open repair of MMC lesions has been used to minimize SCI and neurological deficits before birth. Better cellular and molecular insight into neurological disfunction of MMC may lead to more effective targeted therapies.

## Neuronal Regeneration and Degeneration After Retinal Ganglion Cell (RGC) Injury

Mature CNS neurons, including RGCs, fail to regenerate after axotomy largely because of their reduced intrinsic growth capacity and the extrinsic nonpermissive environment around the lesion. Recent studies have advanced this important research area with ONC models. Yang et al. reviewed recent progress in identifying effective strategies for promoting long-distance regeneration of injured optic nerves and subsequent reconnections to central neuronal targets for potential functional recovery. Combinatory manipulation of multiple crucial genes and pathways could stimulate long-distance axon regeneration in mice with ONC. Proper guidance of regenerating RGC axons to reach their original targeted nuclei in the brain is essential for visual functional recovery. After regenerating axons reach the brain targets, their remyelination and formation of useful synaptic reconnections with targeted neurons are crucial for restoring visual function. So far, several studies have reported long-distance axon regeneration and partial visual function restoration in animal models. Sergeeva et al. summarized recent understanding of the function of interneurons in regulating RGC survival and axon regeneration after ONC. In addition to the intrinsic mechanisms, neurodegeneration and regenerative failure of axotomized RGCs are influenced by the extracellular environment of glial or inflammatory cells. Activity changes of amacrines, a type of inhibitory retinal interneurons, alter the multicellular signaling cascades and contribute to RGC fate, indicating that the complex circuitry in the retina affects RGC survival and axon regenerating ability.

The SWI/SNF complexes (known as BRG1/BRM-associated factor or BAF) are the key regulators of nucleosome positioning, rearrange chromatin structure, and control gene expression and cell proliferation. Deletion of Arid1a, one major component of SWI/SNF complexes, promotes regeneration of multiple tissues in mammals. Peng et al. reported that Arid1a deletion did not stimulate regeneration of injured optic axons, but significantly enhanced RGC survival in mice with ONC. Transcriptome assay displayed alterations of apoptosis-related genes and the JAK/STAT signaling pathway. Thus, Arid1a appears to regulate degeneration of axotomized RGCs. To detect effective assays for monitoring progression of RGC degeneration after ONC, Li, Huang et al. correlated RGC histological data with the results of several optical tests in mice, including scanning laser ophthalmoscopy, optical coherence tomography, and pattern electroretinogram at different time points after ONC. Scanning laser ophthalmoscopy fundus imaging directly visualized the RGC morphology and correlated best with histological quantification of RGC somata and optic axons. Optical coherence tomography detected substantial retinal swelling at the early time points 1–5 days after ONC and the apparent retinal thinning at late time points of 14 and 28 days after ONC. Pattern electroretinogram seems highly sensitive for detecting the early RGC functional deficit before substantial RGC loss, but the limited progression in functional loss may prevent its use as a reliable indicator for RGC degeneration.

## Therapeutic Targets in Neurodegenerative Diseases

Mitochondria supply chemical energy of ATP and are essential for maintaining metabolism, intracellular ionic homeostasis, and other critical functions of cells. Their dysfunction contributes to neural damage and pathophysiology of numerous diseases. Yan et al. highlighted the contribution of mitochondrial dysfunction to pathophysiology of several neurodegenerative diseases, including Alzheimer's, Parkinson's, Huntington's, and amyotrophic lateral sclerosis. Impaired mitochondria, aberrant mitochondrial quality (e.g., imbalanced mitochondrial dynamics, size, and morphology), and mitochondrial-based bioenergetic defects contribute to neurodegeneration and cell loss in many neurological diseases. Recent investigations have advanced our understanding of the molecular basis for mitochondrial impairment and mitochondrial-driven inflammation in the lesioned CNS. Some studies uncovered novel and promising therapeutic targets for neurological diseases.

Pericytes are the multi-functional mural cells of microcirculation that control blood flow, vascular permeability, and homeostasis. Pericytes respond to CNS injury and inflammation by restricting blood capillaries, modulating immune responses, contributing to blood-brain barrier (BBB) disruption and tissue fibrosis, and regulating the repair process of blood vessels. Pericytes represent promising cellular targets for designing therapies for CNS disorders. Laredo et al. reviewed the potential functions of pericytes on regulating angiogenesis, BBB integrity, neurodegeneration, neuroinflammation, tissue fibrosis, and axon regeneration failure after CNS injury or in neurodegenerative diseases, including Alzheimer's and diabetic retinopathy. This review also outlined the strategies to repair injured CNS by targeting pericytes.

Epilepsy, a brain disorder that causes seizures, has the potential to cause serious neurological consequences, including unnecessary bodily injury, psychological and psychiatric impairment, social disability, and diminished lifespan. Because of the restrictions of currently available drug and surgical treatments (e.g., drug resistance, serious side effects, and recurrence), it is interesting to identify more effective therapies for epilepsy. Zhu et al. reported the neuroprotection by treatment with xenon, a gas chemical element found in Earth's atmosphere, using an acute rat seizure model induced by kainic acid. The increased autophagy was probably attributed to the neuroprotection by xenon.

Radiotherapy of the brain malignancies frequently results in persistent cognitive dysfunction in most patient survivors. It is crucial to find the strategies to reverse radiotherapy-induced neurological deficits. Reduced neurogenesis and microvascular integrity, impaired synaptic plasticity, increased inflammation, and other neural alterations probably contribute to radiotherapy-caused brain dysfunctions in animal models. Parihar et al. reported that treatment with AM251, a cannabinoid receptor-1 antagonist, improved hippocampal neurogenesis and reduced expression of proinflammatory markers in mice that received cranial irradiation. This treatment also improved the memory and mood symptoms in the mouse model.

## Therapeutic Potential of Reprogramming Glial Cells Into Neurons and Use of Cerebral Organoid Models for Neural Repair Research

Because of the regeneration failure of injured CNS neurons, many researchers attempted to reprogram resident glial cells into functional neurons for neural repair. Numerous studies reported reprogrammed neuronal progenitors and neurons in the CNS of adult mammals. Tai et al. updated the progress in innate adult neurogenesis under pathological conditions by cell fate reprogramming. This review summarized the major transcriptional factors used for glial reprogramming and the potential of fate reprogramming-based regeneration for treating SCI and other neurological diseases. Despite the controversies on this topic (see Wang et al., 2021), some researchers reported the direct conversion of reactive glial cells into functional neurons in adult mouse brains by expressing the single neural transcription factor of NeuroD1 in astrocytes. Zhang et al. reported the glia-to-neuron conversion by AAV-mediated transduction of NeuroD1 in mice with a cortical stab injury. AAV-NeuroD1 application converted reactive astrocytes into neurons both before and after glial scar formation and the remaining astrocytes proliferated to repopulate themselves. The newly formed neurons appeared to function well; they fired action potentials and established synaptic connections with other neurons. Also, NeuroD1 expression in reactive astrocytes reduced the A1 toxic subtype of astrocytes and reactive microglia and neuroinflammation.

Human cerebral organoid, a three-dimensional cell culture system, recapitulates the developing human brain and is a valuable tool for studying the pathology and treatments of neurological disorders. But cerebral organoids have various shortcomings, including variability, reproducibility, and underrepresenting certain cell types of the brain. Bodnar et al. reported a new scalable and simplified system to generate microglia-containing cerebral organoids, which harbor various neural cells (microglia, astrocytes, neurons, and neural stem/progenitor cells) and mature in a manner that resembles brain development. This group directly transitioned 3D cultures of inducible pluripotent stem cells to embryoid bodies and simplified the steps to generate cerebral organoids.

In Sum, the articles in this Research Topic cover recent interesting findings in CNS neural repair, regeneration, and plasticity using various cell culture, traumatic, and neurodegenerative model systems. We hope that the research advances on this topic will provide important molecular and cellular insight into the complicated CNS lesions and facilitate the development of novel and highly effective therapeutic strategies for neurological diseases, including TBI and SCI. Future studies on the covered topic may validate the effective strategies in primates and humans, develop optimal strategies to target diverse mechanisms for CNS damages and identify additional genes and cell components for neural repair, appropriate targets of reconnected neurons, and synaptic specificity for targeted functional recovery. As the guest editors, including Drs. Andrea Tedeschi (Ohio State University) and Junfang Wu (University of Maryland), we are very grateful to all authors for their invaluable contributions. We hope that all our readers enjoy the review and original articles published on this Research Topic.

## Author Contributions

The author confirms being the sole contributor of this work and has approved it for publication.

## Conflict of Interest

The author declares that the research was conducted in the absence of any commercial or financial relationships that could be construed as a potential conflict of interest.

## Publisher's Note

All claims expressed in this article are solely those of the authors and do not necessarily represent those of their affiliated organizations, or those of the publisher, the editors and the reviewers. Any product that may be evaluated in this article, or claim that may be made by its manufacturer, is not guaranteed or endorsed by the publisher.
